# High-throughput continuous-flow microfluidic electroporation of mRNA into primary human T cells for applications in cellular therapy manufacturing

**DOI:** 10.1038/s41598-020-73755-0

**Published:** 2020-10-22

**Authors:** Charles A. Lissandrello, Jose A. Santos, Peter Hsi, Michaela Welch, Vienna L. Mott, Ernest S. Kim, Jordan Chesin, Nerses J. Haroutunian, Aaron G. Stoddard, Andrew Czarnecki, Jonathan R. Coppeta, Daniel K. Freeman, Deborah A. Flusberg, Jenna L. Balestrini, Vishal Tandon

**Affiliations:** The Charles Stark Draper Laboratory, Inc., Cambridge, MA 02139 USA

**Keywords:** Lab-on-a-chip, Gene delivery, Transfection, Gene therapy, Cancer immunotherapy, T cells, Biomedical engineering

## Abstract

Implementation of gene editing technologies such as CRISPR/Cas9 in the manufacture of novel cell-based therapeutics has the potential to enable highly-targeted, stable, and persistent genome modifications without the use of viral vectors. Electroporation has emerged as a preferred method for delivering gene-editing machinery to target cells, but a major challenge remaining is that most commercial electroporation machines are built for research and process development rather than for large-scale, automated cellular therapy manufacturing. Here we present a microfluidic continuous-flow electrotransfection device designed for precise, consistent, and high-throughput genetic modification of target cells in cellular therapy manufacturing applications. We optimized our device for delivery of mRNA into primary human T cells and demonstrated up to 95% transfection efficiency with minimum impact on cell viability and expansion potential. We additionally demonstrated processing of samples comprising up to 500 million T cells at a rate of 20 million cells/min. We anticipate that our device will help to streamline the production of autologous therapies requiring on the order of 10$^8$–10$^9$ cells, and that it is well-suited to scale for production of trillions of cells to support emerging allogeneic therapies.

## Introduction

Recent breakout successes in adoptive cell transfer based immunotherapies have revitalized the field of cellular therapy and greatly increased demand for improved cell bioprocessing and gene delivery technologies^1^. In particular, the FDA has granted approval for the use of T cells modified to express chimeric antigen receptor (CAR) genes for treatment of certain hematological cancers^2–6^. The manufacturing chain for CAR T-cell based therapeutics currently relies mainly on lentiviral-based transduction for gene delivery^1,7^, but these vectors are complex and expensive to manufacture^8^, have limited payload capacity^9^, and carry safety concerns because they integrate genetic information into the genome in an uncontrolled way^10^. Although some progress has been made to reduce costs by improving viral transduction efficiency^11^, additional tools will be necessary to address all of these challenges. Researchers have explored gene-editing tools such as CRISPR/Cas9 combined with alternative gene delivery methods^12^ including electrotransfection^13^, lipofection^14,15^, and physical membrane deformation^16–19^. Electrotransfection in particular has been demonstrated in several cellular therapy manufacturing workflows, including direct delivery of guide RNA (gRNA) complexed with Cas9 protein in the form of a ribonucleoprotein (RNP) complex for gene disruption^20^, delivery of mRNAs that encode for gene-editing proteins^21^, delivery of multiple plasmids as transposon/transposase systems^22^, and co-electroporation of RNPs with a double-stranded DNA (dsDNA) homology-directed repair (HDR) template for totally non-viral, targeted gene insertion^13^.

Most commercial electroporation machines, however, are built for research use and process development rather than for large-scale clinical biomanufacturing, and therefore lack the precision, automation, throughput, and integration potential needed to scale up cellular therapy production. Several microfluidic approaches have been developed to attempt to address these shortcomings^23,24^. They offer: (1) more precise control of electric field exposure, a feature which is key to achieve efficient transfection while maintaining high cell viability^25^, (2) favorable thermal characteristics to efficiently remove heat which can harm nearby cells^24^, and (3) continuous-flow, semi-automated processing, a key feature for future process integration. The major challenge for microfluidic approaches is scaling the technology to meet growing clinical throughput needs, where on the order of 10$^8$–10$^9$ cells per dose are needed for many autologous therapies^3,26^, and rapid processing of on the order of 10$^{12}$ cells is expected to be needed for emerging allogeneic therapies^27,28^. Here we present a novel microfluidic continuous-flow electrotransfection device designed for precise, consistent, and high-throughput genetic modification of target cells for cellular therapy manufacturing applications. We optimize our device and process for delivery of mRNA to primary human T cells and demonstrate efficient genetic modification of samples comprising up to 500 million T cells with minimum impact on cell viability and expansion potential. This is an important application of electrotransfection, as delivery of mRNA encoding for a therapeutic gene results in transient gene expression, which avoids genotoxicity and DNA toxicity but can still produce an antitumor effect^29^. Furthermore, delivery of mRNA encoding for gene-editing tools such as transcription activator-like effector nucleases (TALENs) can generate stable and permanent changes to the genome^21^. Our data demonstrates the potential of our system to efficiently deliver mRNA to primary human T cells and provides a foundation for future efforts which may focus on optimizing delivery of additional payloads and on the increased throughput needs of allogeneic therapies.

## Methodology

### System overview

**Figure 1:**
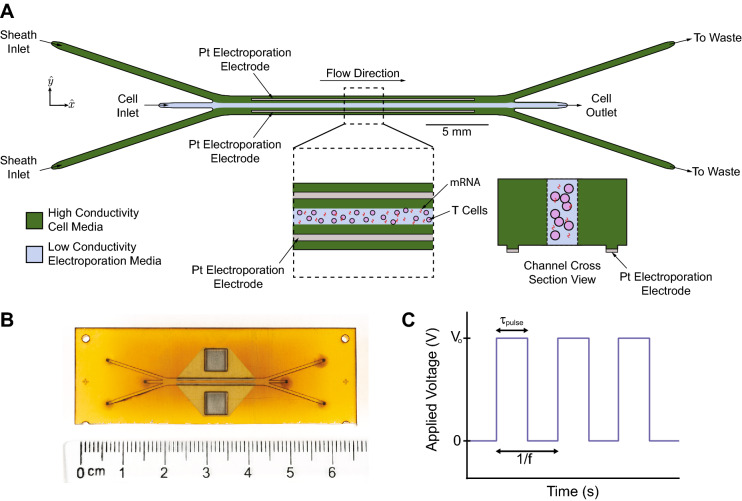
Our microfluidic, continuous-flow electrotransfection device. (**A**) Scheme of our device. High-conductivity cell culture medium (RPMI in this study) is introduced into the sheath inlets, and low-conductivity electroporation medium (BTXpress in this study) with cells and transfection payload are introduced into the central cell inlet. As cells flow through the device, platinum electrodes in contact with the sheath streams are energized to deliver pulsed electric fields. (**B**) Photograph of our microfluidic electrotransfection device. (**C**) Scheme of the voltage waveform that is applied to the electrodes for transfection, with important parameters highlighted. In this study, monophasic square-wave pulse trains are applied with a peak voltage of $\text {V}_o$, a pulse duration of $\tau _{\text {pulse}}$, and a pulse frequency or repetition rate of *f*.

Our microfluidic device continuously and consistently delivers electrical pulses across a stream of cell- and payload-laden media in order to achieve efficient electrotransfection of cells (Fig. 1). The device comprises a stack of precision-laser-cut layers (Figure S1) of polyetherimide (PEI) sheets that form a microfluidic channel of rectangular cross-section. The channel has trifurcations at both ends to support the generation of a stable sheath flow. The straight portion of the channel is 1.5 mm in width, 0.25 mm in height, and 21 mm in length. Aqueous media and cell suspensions, driven by a syringe pump, enter the channel at the trifurcated inlet, travel in the $\hat{x}$-direction, and leave the channel at the trifurcated outlet. The flow is laminar for the parameters explored in this study (Reynolds number $\sim 10$), leading to diffusion-dominated mixing across fluid streamlines. Cells and media enter and leave the microchannel through holes in the floor of the channel at the upstream and downstream ends, respectively. Stainless steel (SS) tubing is inserted, fixed in place, and fluidically-sealed with epoxy at the inlets and outlets.

In typical practice (Figure S2), the central sheath stream comprises cells and payload suspended in low-conductivity electroporation media, and the two side streams surrounding the center flow contain high-conductivity cell culture media. Two rectangular, platinum, thin-film electrodes are sputter-deposited on the floor of the channel near the side walls, and are connected to a custom electrical pulse waveform generation system by soldered wire leads. The custom system can generate arbitrary waveforms; in this study we focused on monophasic pulse trains parameterized by a pulse voltage, $\text {V}_o$, a pulse duration, $\tau _{\text {pulse}}$, and a pulse frequency, *f* (Fig. 1C). These parameters are tuned to control the total electric field dose per pulse and the number of pulses delivered, on average, per cell residence time in the channel. The patterned electrodes are rectangular in geometry (18 mm length and 150 μm width) and are positioned such that they make contact only with the sheath fluid. This configuration is advantageous for several reasons: (1) it enables a concentration of the electric field across the width of the low-conductivity media, with negligible voltage drop across the high-conductivity buffer, and (2) it prevents the cells from making physical contact with the electrodes and the sidewalls of the channel, keeping them away from regions of local electric field concentration and from potentially cytotoxic electrochemical reaction byproducts. This aids in maintaining high cell recovery and viability. This type of flow configuration has been used successfully in the past to transfect HEK-293A, HeLa, neuro-2A, and HEK-293 mammalian cell lines^30^ and yeast cells^31^, and by our group to deliver mRNA to primary human T cells^32^. Compared with these previous efforts, our device is designed for orders-of-magnitude greater throughput for clinical-scale processing (enabled by increased channel cross-sectional dimensions and a sturdier material set), is fabricated from hard plastics compatible with the transition to mass-manufacturing, and has been optimized for primary human T cells rather than model mammalian cell lines. Our device also improves upon our own previously-demonstrated transfection performance^32^ in terms of electroporation efficiency.

### Performance metrics

In this manuscript, we report transfection efficiency and viability as key indicators of the performance of our electroporation system. To measure transfection efficiency, we delivered mRNA that encoded a fluorescent reporter protein (CleanCap mCherry mRNA, TriLink Biotechnologies, San Diego, CA), then measured expression of the protein by flow cytometry 24 h after transfection. We simultaneously measured viability using Sytox live/dead stain (ThermoFisher Scientific, Waltham, MA). Transfection efficiency is defined as:

1 $$\begin{aligned} \text {Transfection Efficiency }(\%) = \frac{\text {N}_{\text {expressing}}}{\text {N}_{\text {viable}}} \times 100, \end{aligned}$$

where $\text {N}_{\text {expressing}}$ is the number of cells expressing the reporter protein 24 h after transfection in our device, and $\text {N}_{\text {viable}}$ is the total number of viable cells at the same time point. Viability is reported as a ratio of the viability measured 24 h after transfection relative to the initial viability, rather than the absolute viability, to account for natural donor-to-donor varibility in the initial product. Typical starting values for absolute viability were in the range 85–90% for all experiments. The viability ratio is defined as:

2 $$\begin{aligned} \text {Viability Ratio} =\frac{\text {V}_{\text {eluted}}}{\text {V}_{\text {i}}}= \frac{1}{\text {V}_{\text {i}}}\cdot \frac{\text {N}_{\text {viable}}}{\text {N}_{\text {eluted}}}, \end{aligned}$$

where $\text {V}_{\text {i}}$ is the initial viability of the cells introduced into the device and $\text {V}_{\text {eluted}}$ is the viability of the cells eluted from the device, as measured 24 h after transfection. $\text {N}_{\text {eluted}}$ is the total number of cells eluted from the device, including both live and dead cells, counted 24 h after transfection. A typical viability measurement, however, does not account for cells that were potentially obliterated or lost during electrotransfection. Therefore, we also include coarse estimates of recovery, defined as:

3 $$\begin{aligned} \text {Recovery }(\%) = \frac{\text {N}_{\text {eluted}}}{\text {N}_{\text {input}}} \times 100, \end{aligned}$$

where $\text {N}_{\text {input}}$ is the total number of cells that were introduced into the electrotransfection device, estimated from the product of the collection time, input cell concentration, and input cell flow rate. Because estimation of recovery requires accurate cell counts of both the input and output samples, it is a challenging and noisy measurement, and is often not reported.

## Results

### Simulation of electric fields generated in the device

**Figure 2:**
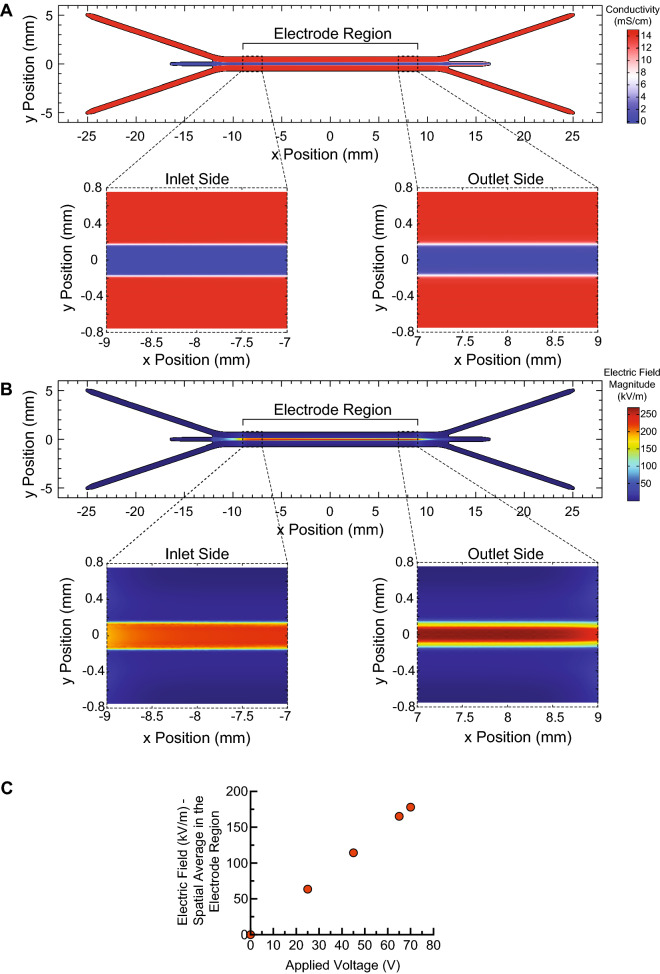
Simulations of electric fields generated in our device as a function of applied voltage. (**A**) A combination of convective transport due to imposed flow and diffusive mixing contribute to the steady-state fluid conductivity distribution in our device. High-conductivity RPMI ($\sigma$ = 14 mS/cm) is introduced into each side inlet at 550 μl/min, and low-conductivity BTXpress electroporation media with cells and mRNA ($\sigma \approx$ 0.5 mS/cm) is introduced into the center stream. The conductivity of the cell stream increases toward the device outlet due to infiltration of ions from the side streams by diffusion. The midplane in channel depth is shown. (**B**) Electric field magnitude distribution that results when 70 V is applied to the electrodes. Due to the conductivity distribution, the electric field magnitude is much higher in the cell stream. Mixing of the high and low-conductivity streams due to diffusion compresses and intensifies the high-field region near the outlet. (**C**) Spatially-averaged electric field magnitude in the cell stream as a function of applied voltage. (**A**) and (**B**) were generated using COMSOL Multiphysics software and (**C**) was generated using GraphPad Prism software.

Electric field magnitudes generated in our device were estimated using a finite element analysis (FEA) model implemented in COMSOL Multiphysics software (see Supplementary Methods). The trifurcating inlets and outlets in our sheath flow device were designed to support a side-to-center flow ratio of approximately 2.75:1. In this study, we introduced high-conductivity RPMI media ($\sigma \approx$ 14 mS/cm) into the side inlets, each with a flow rate of 550 μl/min. We introduced cells and mRNA suspended in low-conductivity BTXpress electroporation media ($\sigma \approx$ 0.5 mS/cm) into the center inlet at a flow rate of 400 μl/min. These flow conditions were used for all experiments reported in this study, and were also assumed for simulations. At this flow ratio, our simulations predict that the center stream width is approximately 360 μm, though diffusion between the low and high-conductivity streams blurs the interface between the different media near the outlets (Fig. 2A). We used a custom, in-house built amplifier system to energize the electroporation electrodes, which was capable of generating a maximum of 70 V. At this voltage, the volumetric average of the electric field magnitude within the central fluid stream was calculated to be 178 kV/m in the electrode region (Fig. 2B). Diffusive mixing between the side and center streams creates spatial non-uniformities toward the outlet, amplifying the electric field magnitude at the center of the channel width to a maximum of 282 kV/m, and reducing it on the edges of the center stream. As expected, the electric field magnitude is linearly proportional to the applied voltage (Fig. 2C). We report data throughout this manuscript in terms of the spatially-averaged electric field magnitude, as shown in Fig. 2C.

### Effect of mRNA concentration on transfection efficiency

**Figure 3:**
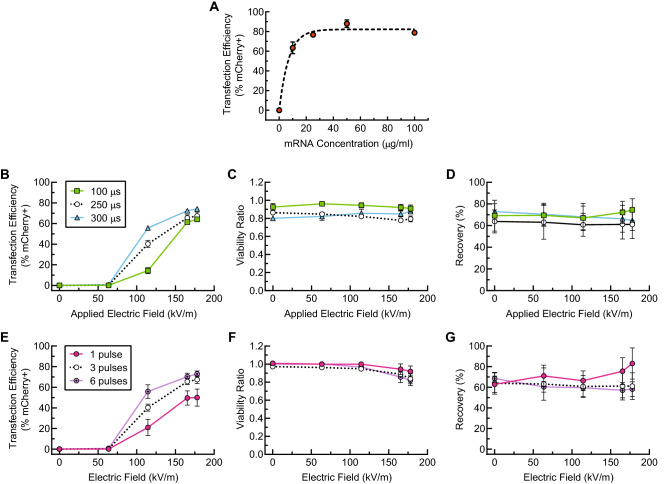
Parametric study of transfection of resting primary human T cells from three independent, healthy donors (n = 3) with mCherry-encoding mRNA using our device. For all of these data, cells were suspended in BTXpress electroporation medium at a concentration of 2 million/ml. (**A**) Transfection efficiency in our device as a function of mRNA concentration. The applied Voltage, $V_o$, was 65 V (corresponding to an electric field magnitude of 165 kV/m); the pulse duration, $\tau _{\text {pulse}}$, was 250 μs; the pulse frequency, *f*, was 10 Hz (approximately 3 pulses per cell residence time in the device). An exponential fit is shown to help guide the eye. (**B**–**D**) show the transfection efficiency, viability ratio, and recovery, respectively, for a parameter sweep in which the pulse frequency, *f*, was held fixed at 10 Hz (approximately 3 pulses per cell residence time). The applied voltage, $V_o$, was varied from 0 to 70 V (Electric field magnitude ranging from 0 to 178 kV/m), and pulse durations of 100, 250, and 300 μs were tested. (**E**–**G**) show transfection efficiency, viability ratio, and recovery, respectively, for a parameter sweep in which the pulse duration, $\tau _{\text {pulse}}$, was held fixed at 250 μs. The applied voltage, $V_o$, was varied from 0 to 70 V, and pulse repetition rates of 3.3, 10, and 20 Hz corresponding to approximately 1, 3, and 6 pulses per cell residence time were tested. All data presented are an average of replicates from three independent, healthy donors and error bars represent standard error of the mean. Some error bars are too small to be visible. This figure was generated using GraphPad Prism software.

We performed an mRNA dose-response experiment to optimize mRNA concentration for maximum transfection efficiency using as little mRNA as possible. For these experiments, the pulse repetition rate, pulse width, and pulse magnitude were fixed such that cells were exposed to three electrical pulses (10 Hz repetition rate and 400 μl/min cell media flow rate) with a monophasic square-wave pulse of duration $\tau$ = 250 μs and an amplitude of 65 V (approximate electric field strength of 145 kV/m). Unstimulated primary human T cells from three independent, healthy donors were suspended in BTXpress low-conductivity electroporation media at a concentration of 2 million/ml and mCherry-encoding mRNA was added at concentrations ranging from 0 to 100 μg/ml. All samples were electroporated using the same electroporation parameters including the 0 μg/ml sample. Transfection efficiency increased from 0% to approximately 80% with increasing mRNA concentration from 0 to 100 μg/ml (Fig. 3A). For this cell concentration, the dose-response curve reached saturation at an mRNA concentration of 25–30 μg/ml. To ensure adequate sensitivity to changes in electric field exposure, subsequent electrical parameter optimization experiments were carried out at a sub-saturating mRNA concentration of 20 μg/ml.

### Optimization of electrical parameters

To determine optimal electrical parameters for electrotransfection of mRNA into primary human T cells for our device, we tested the effects of varying electric field strength, pulse width, and pulse number on transfection efficiency, viability, and recovery. For all of these experiments, unstimulated T cells (from three independent, healthy donors) and mCherry-encoding mRNA were suspended in BTXpress low-conductivity electroporation buffer at final concentrations of 2 million/ml and 20 μg/ml, respectively, and flowed into the center channel of the device at a rate of 400 μl/min. Electrical pulses were applied to the cells at varying voltages, pulse durations, and pulse repetition rates. When pulse repetition rate was kept constant (10 Hz, corresponding to approximately 3 pulses per average cell residence time in the active field region of the microchannel), transfection efficiency increased both with increasing electric field strength and with increasing pulse duration (Fig. 3B). We observed a strong threshold effect with the electric field magnitude, where transfection efficiency was essentially zero for tested field strengths of 64 kV/m (25 V applied) and below. Transfection efficiency increased with increasing electric field magnitude above 64 kV/m, reaching a plateau of approximately 70% at about 165–178 kV/m (65–70 V) for all of the pulse widths tested. At the longest pulse width (300 μs), higher efficiency was observed at lower field magnitudes compared to shorter pulse widths, with transfection efficiency reaching nearly 60% at 114 kV/m (45 V), compared to only 15% for a 100-μs pulse width. For each of the three pulse widths tested, viability remained high and relatively constant (80–95% of the initial viability) for all of the electric field magnitudes applied (Fig. 3C). Viability was 10–15% lower for the longer pulse widths (250 and 300 μs) than it was for the 100-μs pulse width.

When pulse duration was kept constant at 250 μs and the approximate number of pulses applied per average cell residence time was varied between 1, 3, and 6, transfection efficiency increased with increasing numbers of pulses (Fig. 3E). As with pulse duration, the largest gains in transfection efficiency were observed at the 114 kV/m field strength, at which the transfection efficiency was nearly 60% when 6 pulses were applied, compared to only 20% when 1 pulse was applied. At the highest fields applied (165–178 kV/m), transfection efficiencies were similar for 3 pulses and for 6 pulses (70%), but were reduced when only one pulse was applied (40–60%). Viability remained high (above 80% of the initial viability) for all the conditions tested (Fig. 3F), but was highest at low field strengths and pulse numbers and declined as both of these parameters increased. At the lower field strengths, viability remained at 95–100% of initial viability regardless of pulse number, but decreased to about 80% as both field strength and pulse number increased. Viability also remained high (90–95%) when applying only 1 pulse at higher field strength (165–178 kV/m).

**Figure 4:**
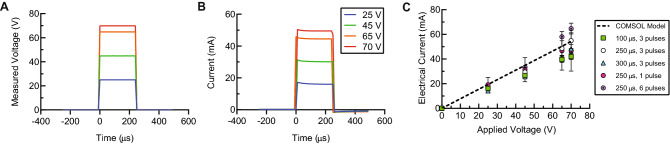
Electrical current and voltage measurements during application of pulsed electric fields enables verification of successful waveform delivery. (**A**) Representative data showing amplified voltage output delivered to the microfluidic device for pulses 250-μs in duration and 25, 45, 65, and 70 V in magnitude. For each voltage, a single pulse is shown. (**B**) Corresponding measured electrical currents for the pulses shown in (**A**). (**C**) The currents measured during the transfection experiments corresponding to the data shown in Fig. 3B–G are plotted against the applied voltage and compared to the COMSOL model prediction. For each donor replicate (n = 3) and each condition, the average peak current over 1 s was measured. The mean of the donor replicates is shown, and error bars represent standard error of the mean. This figure was generated using GraphPad Prism software.

During these experiments, we measured the electrical current flowing through the system while pulses were delivered (Fig. 4). This measurement serves to diagnose whether electric field pulses were successfully delivered to the cells. In the Faradaic regime^33^, the electrical load in our system is almost entirely resistive, so the electrical current waveforms are expected to be linearly proportional to the applied voltage waveforms, which was observed in all experiments (Fig. 4A,B). Furthermore, the measured peak currents as a function of applied voltage agree well with those predicted by our computational model (Fig. 4C) with a slight increase, relative to the model, observed for the measurements taken at the highest repetition rate (6 pulses) and applied voltages. We hypothesize that this increase in current was caused by a decrease in the bulk fluid resistivity due to Joule heating.

### Effects of density of cells in suspension on transfection efficiency

**Figure 5:**
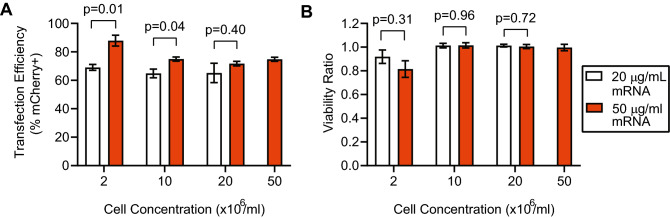
Device throughput can be increased by running with a higher cell concentration. Transfection efficiency is presented for resting T-cell concentrations ranging from 2 to 50 million/ml, and mCherry-encoding mRNA concentrations of 20 μg/ml and 50 μg/ml. High transfection efficiency (70–85%) is maintained at cell concentrations up to 50 million/ml. At the highest cell concentration tested, the device processed approximately 20 million cells/min. The data for 50 million cells/ml was only run with the 50 μg/ml mRNA concentration. These data are an average of replicates from three independent, healthy donors (n = 3). The same three donors were used for experiments run at 2, 10, and 20 million cells/ml, and a new set of three donors was used for experiments run at 50 million cells/ml. Error bars represent standard error of the mean. This figure was generated using GraphPad Prism software.

We next examined the effect of increasing the concentration of cells in the suspension introduced into our device on transfection efficiency (Fig. 5). Successful transfection of cells at high cell concentrations is critical for increasing throughput to a clinically-relevant scale. We anticipated that transfection at higher cell concentrations might require an attendant increase in mRNA concentration to maintain transfection efficiency. Therefore, for these experiments, suspensions of unstimulated primary human T cells in BTXpress electroporation media were prepared at 2 million, 10 million, 20 million, and 50 million cells/ml. T cell suspensions were mixed with mCherry-encoding mRNA at concentrations of 20 or 50 μg/ml (the 50 million cells/ml suspension was only tested with an mRNA concentration of 50 μg/ml due to available resources). The suspensions were flowed into our device at 400 μl/min, and electroporated with approximately 3, 250-μs electric field pulses at 165 kV/m (65 V). This flow rate and range of cell concentrations corresponds to a total processing throughput ranging from 0.8 to 20 million cells/min.

With the 50 μg/ml mRNA concentration, transfection efficiency was similar for the cell suspensions at 10, 20, and 50 million/ml (72–75%), and was higher for cells suspended at 2 million/ml (88%). Decreasing the mRNA concentration to 20 μg/ml resulted in a decrease in transfection efficiency for the suspensions at 2, 10, and 20 million cells/ml, but the decrease was only statistically significant for the suspensions at 2 and 10 million cells/ml. Viability was similar for all of the conditions tested, and greater than 90% of the initial viability for all conditions except for the 2 million cells/ml and 20 μg/ml mRNA case (82%). There were no statistically significant differences in viability for suspensions tested with an mRNA concentration of 20 μg/ml vs. the 50 μg/ml concentration.

### Post-transfection expansion and expression over time

**Figure 6:**
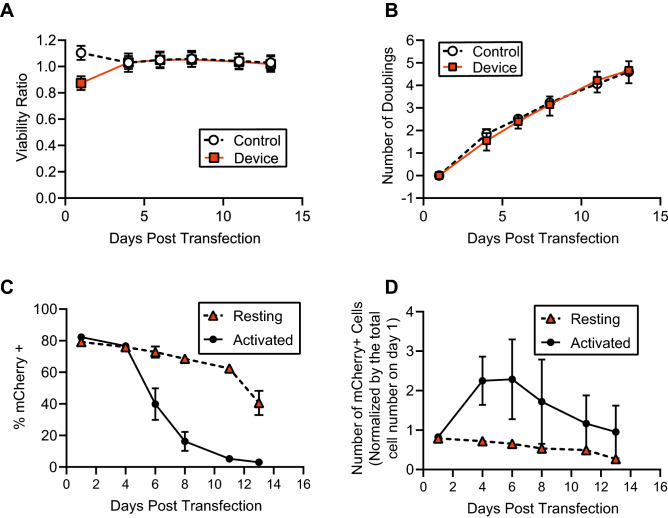
Long-term effects of continuous-flow microfluidic electrotransfection on T-cell viability and expansion rate. T cells were transfected with mRNA on Day 0. (**A**) Viability of activated, expanding T cells over a period spanning 13 days normalized to the pre-transfection viability on Day 0. After a short-term drop in viability by 5–13% 24 h after transfection, viability remained stable and similar to that of control cells that were not transfected or introduced into our device. (**B**) Population doubling times for transfected cells were nearly indistinguishable from control cells. (**C**) Comparison of mCherry expression over time post transfection between activated T cells and resting T cells. (**D**) Comparison of the total number of mCherry-expressing cells over time post transfection between activated T cells and resting T cells. The total cell number is normalized to the total number of cells on day 1 (24 h after transfection). All data presented are an average of replicates from three independent, healthy donors (n = 3) and error bars represent standard error of the mean. Some error bars are too small to be visible. This figure was generated using GraphPad Prism software.

To assess the long-term effects of microfluidic electrotransfection using our device, we monitored cell viability, mCherry expression, and growth rate of activated (expanding) primary human T cells for 13 days following transfection with mRNA (Fig. 6). We used activated T cells in these experiments in order to monitor cell growth over time, since resting (unstimulated) cells used in our previous experiments are generally not dividing. The viability of the activated cells dropped by 5–13% 24 h after transfection, but recovered to that of the initial sample within 4 days post transfection (Fig. 6A), and remained at that level for the duration of observation. Moreover, the rate of growth for transfected cells was nearly indistinguishable from that of control cells that were never introduced into our device (Fig. 6B). For both transfected and control cells, the cell population doubled approximately every 2.6–2.7 days. In addition, we compared mCherry expression over the same time period in activated (stimulated for 48 h prior to transfection) vs. unstimulated resting T cells. The percentage of cells expressing the mCherry reporter gene declined over time for both activated and resting T cells (Fig. 6C), but declined much more rapidly for the activated cells starting 4–6 days after transfection. After 13 days of observation, the fraction of resting cells expressing mCherry decreased from approximately 80% to 40%, whereas in activated T cells mCherry expression after 13 days was reduced to 3%. However, due to the expansion of the activated T cells, the total number of activated T cells expressing mCherry increased by a factor of $\sim$ 2 from Day 1 to Day 4, and then declined monotonically up to Day 13 (Fig. 6D). The total number of resting T cells expressing mCherry declined over time from Day 1 to Day 13 monotonically, corresponding with the observed decline in the percentage of the population expressing the reporter. A more detailed analysis of the differences in expression between the resting and activated cells can be found in the Discussion section below.

### Clinical-scale processing: continuous-flow transfection of $\sim$ 500 million T cells

**Figure 7:**
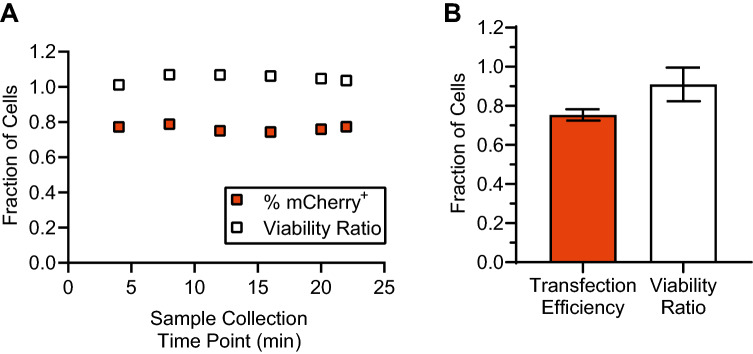
Our device is consistent and effective when used for transfection of clinically-relevant numbers of cells (approximately 200 million–500 million cells per donor). (**A**) Representative data from one primary human T-cell donor. All of the T cells from one buffy coat donor (approximately 500 million cells total) were activated, resuspended in BTXpress electroporation media at 50 million cells/ml, and then transfected with mCherry-encoding mRNA (50 μg/ml) continuously in a single microfluidic electroporation channel over a time span of approximately 20 min. Samples taken at approximately 4-min intervals show that transfection efficiency and viability (measured 24 h post-transfection) are consistent throughout processing. (**B**) T cells were isolated from buffy coat samples from three independent, healthy donors and activated. Each donor produced approximately 200 million–500 million T cells. The entirety of each donor sample was then transfected with mCherry mRNA. Average transfection efficiency and viability ratio across the three donors is shown (n = 3), where error bars represent standard error of the mean. This figure was generated using GraphPad Prism software.

One of the major limitations of traditional cuvette-based electroporation systems is that they require batch transfection of cells with limited numbers of cells per batch ($\sim$ 10–50 million cells). Because clinical doses of autologous cellular therapies typically require 100s of millions or billions of cells, we used our device to demonstrate truly continuous transfection of T-cell samples ranging from 200 million to 500 million cells per donor. For these studies, T cells isolated from three independent, healthy human donors were stimulated for 48 h and then resuspended in BTXpress electroporation media at 50 million/ml with 50 μg/ml mCherry-encoding mRNA. These samples were transfected in our device using a monophasic pulse train waveform ($V_o$ = 65 V, *f* = 10 Hz, and $\tau$ = 250 μs). In this configuration, continuous transfection of 500 million T cells was accomplished in 25 min. To test the quality of output from our device over time, we collected output samples approximately every 4 min and measured mCherry expression and viability of each sample 24 h post-transfection for one representative buffy coat donor that produced 500 million cells (Fig. 7A). The average transfection efficiency across all of the collected samples was 75.5%, with a coefficient of variation of 2%. The viability was not reduced compared to the input sample, and also varied by approximately 2%. Across the three buffy coat donors, the mean transfection efficiency was 75%, and the mean viability was 91% of the initial viability (Fig. 7B).

## Discussion

### Effects of mRNA concentration on transfection efficiency

Following permeabilization of cells by an applied electric field, transport of small-molecule cargo into the cells has been described as predominantly driven by diffusion due to the concentration gradient between the inside and outside of the cell^25,34,35^. For larger molecules, like pDNA, electrophoretic transport mechanisms also putatively contribute to transport into the cell^34,36–38^. This has led to many dual or multi-pulse strategies^12^, where one or more high-intensity pulses for permeabilization are followed by a lower intensity, longer pulse for electrophoretic transport. An alternative model is that rather than transporting the DNA across the cellular membrane and through the cytoplasm and nucleus, the electric field may instead serve to associate the DNA with the cell membrane, at which point complex endocytotic or pinocytotic pathways shuttle the DNA into the cell and potentially to the nucleus^25,39–42^. The mCherry-encoding mRNA that served as the test payload for our microfluidic electroporation device (approximately 1 kb) falls between small molecules and typical pDNA vectors in size, and therefore may be transported by a combination of these mechanisms^43^. While the exact mechanisms by which macromolecules are transported into the cell during or following electroporation are not well-understood, all of the proposed mechanisms ultimately indicate that the flux of cargo into the cells is dependent on the probability of cells interacting with the cargo, the number of cargo molecules available per cell, and/or the rate at which cargo is transported from the extracellular environment into the cytoplasm. Thus we expect electrotransfection efficiency to follow a dose-response-like relationship with mRNA concentration. We observed in our device that when applying 3 electric field pulses, each with magnitude of approximately 165 kV/m and a duration of 250 μs, transfection efficiency increased with increasing mRNA concentration, saturating at concentrations above 25 μg/ml when the cell concentration was 2 million/ml. For this reason, we ran subsequent electrical parameter sweeps in our device using an mRNA concentration of 20 μg/ml (below saturation) to ensure that the transfection efficiency was sufficiently sensitive to changes in the pulsed electric field parameters. When electroporating primary murine dendritic cells with eGFP-encoding mRNA using a bulk electroporation system, Van Meirvenne, et al.^44^ observed a similar mRNA-dose dependent transfection efficiency with concentrations ranging from 5 to 100 μg/ml, reaching a maximum in transfection efficiency of approximately 75% at an mRNA concentration of 25 μg/ml. Zhao et al.^45^ did not observe an mRNA dose dependence when electroporating stimulated human peripheral blood lymphocytes (PBLs) with GFP-encoding mRNA, but they tested concentrations that ranged from 50 to 200 μg/ml. This range falls within the saturation region of our data (Fig. 3A). At these high concentrations, they achieved transfection efficiency (96%) and viability (95%) similar to our data using a Harvard Apparatus BTX bulk electroporation system with 4-mm cuvettes.

### Electrical parameters

The classic description of electroporation is that exposing a cell to a pulsed electric field results in the formation of transient aqueous pores within the lipid bilayer of the cellular membrane, through which genetic material or other cargo can be introduced. Exposing a cell to an electric field establishes an induced transmembrane potential^46–48^ and, at some critical value of that potential (typically 0.2 to 1 V^24^), the cellular membrane becomes permeable. The induced potential is proportional to the size of the cell, so larger magnitude electric fields are needed to reach this critical potential in smaller cells^49^. Analytical models, computational models, and molecular dynamics simulations predict that the locations of the permeable regions depend on the cell’s orientation with respect to the electric field^50–52^, which is also observed in experimental data^53,54^. In these models, the degree of permeabilization is also predicted to be proportional to the magnitude of the electrical stimulation. In general, permeabilization is expected to increase with increasing electric field magnitude and duration of exposure, provided that the field magnitude is above the critical threshold value. Increased permeabilization and increased transfection efficiency often come at the cost of decreased cell viability due to irreversible membrane damage, Joule heating, and changes to intracellular salt concentrations^25^.

In our device, we examined how electric field pulse magnitude, pulse duration, and the number of pulses applied affect transfection efficiency of mRNA into primary human T cells (Fig. 3), and the concomitant changes in cell viability and overall cell recovery. As expected, we observed increased transfection efficiency at higher electric field pulse magnitudes and with longer exposure times. There was no transfection observed for field magnitudes of 64 kV/m and below, and transfection efficiency increased with increasing field magnitude in all cases starting at a magnitude of 114 kV/m. This suggests that the critical electric field magnitude is between 64 and 114 kV/m for transfection of mRNA into primary human T cells in our device. Our results are in qualitative agreement with a series of experiments performed by Teissie and Rols^25,47,48^, who also examined the effects of pulse magnitude, pulse duration, and number of pulses in Chinese hamster ovary (CHO) cells using Trypan blue, fluorescent dextrans, and $\beta$-Gal as cargo. They observed a critical electric field of 30 kV/m for CHO cells, which is smaller than what we observed for T cells. This is consistent with the larger size of CHO cells vs. primary human T cells (14–15 μm diameter vs. 7–10 μm) and the relationship between critical electric field and cell diameter predicted by the Schwan equation^49^.

**Figure 8:**
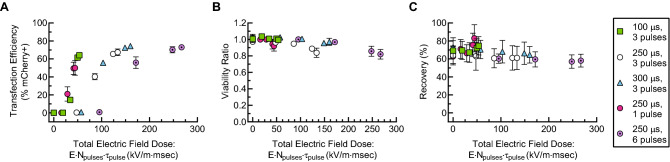
Transfection efficiency (**A**), viability ratio (**B**), and recovery (**C**) as a function of total electric field dose (the product of the electric field magnitude, the number of pulses applied, and the duration of each pulse) for the combined data sets presented in Fig. 3. Primary human T cells were suspended at a concentration of 2 million/ml with 20 μg/ml mCherry-encoding mRNA. Data points represent an average of replicates from three independent, healthy donors (n = 3), and error bars represent standard error of the mean. Some error bars are too small to be visible. This figure was generated using GraphPad Prism software.

To examine our data holistically, we recast it in terms of a total electric field dose (Fig. 8): the product of the applied electric field magnitude and the total time of exposure. This roughly collapses most of the data onto a single curve, in which the transfection efficiency increases with total electric field dose, saturating in the range of 75–80% (Fig. 8A). It is likely that the remaining non-transfected cells did not receive the correct electric field dose due to spatial non uniformities in the electric field. This can be improved through further optimization of device geometry and flow parameters. Furthermore, at the maximum applied voltage tested, we did not observe a large viability drop, so application of higher voltages using a more powerful electrical amplifier may also increase transfection efficiency without leading to excessive cell death. Some of the data does not collapse, as there is a cluster of data points at 0% transfection efficiency. These points represent conditions for which the electric field magnitude was not above the critical threshold value. Thus, these data are consistent with models that predict a critical transmembrane potential that is required for transfection. Viability (relative to the starting cell population viability) decreased linearly with total electric field dose, from nearly 100% to approximately 80% over the range tested (Fig. 8B). Previous studies^25^ indicate that at higher electric field doses, the viability begins to fall off more rapidly and non linearly, though in our study we did not reach these higher doses.

To quantify the importance of each of the electrical parameters studied on transfection efficiency, viability ratio, and recovery, we performed a multifactor analysis of variance (ANOVA) for the data shown in Fig. 3; the results are shown in Table S1. At the 5% significance level, the ANOVA shows that all of the electrical parameters tested impact transfection efficiency, as expected. The choice in T-cell donor, however, did not significantly affect the transfection efficiency. It should be noted, that only healthy T-cell donors were used in this study, and that clinical samples from cancer patients are likely to introduce more variability. It should also be noted that each donor was tested at a different time with a different device, and that the term, “donor,” as used here cannot be decoupled from these other factors. Viability ratio was affected by all of the electrical parameters tested as well as the donor. Recovery is not typically reported for electrotransfection systems, but is critical when the sample of target cells is from a limited source, as is the case for autologous cellular therapies. Recovery is a challenging measurement, because it relies on accurate and sensitive cell counting for a wide dynamic range of cell concentrations. In this study recovery was most strongly affected by the donor (including the other factors as mentioned above), but did not significantly depend on the applied voltage. There was some dependence of recovery on the pulse duration, and the number of pulses, however. Recovery in this data set dropped from about 70% for the smaller electric field doses studied to about 60% for the highest doses studied. We hypothesize that cell sedimentation in the syringe prior to entering the microfluidic device may account for much of the observed cell loss. This may be improved in future studies by introducing a cell agitation mechanism, such as a magnetic stir bar, in the syringe.

### Post-transfection expansion and expression over time

T-cell expansion is highly dependent on the specific culture conditions, the bioreactor, and the culture media used, making direct comparison to expansion data in the literature challenging. Velaga et al.^55^ examined expansion of regulatory T cells (CD8$^{-}$CD19$^{-}$CD25$^{+}$) in several different medias, including TexMACS. They observed a large variation in fold expansion after 14 days of culture, ranging from about 2 to 200. Our expansion rates for both untouched and electrotransfected cells fall within this range, as we observed an average of a 25-fold increase in cell number in approximately the same time period. Hedfors et al.^56^ studied T cells positively selected for CD4 and CD8, and reported a 200–500 fold expansion when cells were grown in RPMI 1640 for 14 days. Given the variabilities in T-cell expansion rates related to culture conditions, it is most meaningful to compare expansion rates for transfected and untouched cells cultured under the same conditions. In this study, we observed no difference in expansion rates for T cells transfected in our device when compared to control cells that were never introduced into a device.

After transfection, the fraction of mCherry-expressing cells declined over time for both resting and activated (expanding) T cells, but it declined more rapidly for the activated cells (Fig. 6C). This is likely due to a combination of two factors: division of mCherry protein to daughter cells, in a manner similar to that observed for cell-tracking dyes^57^, and potential differences in protein turnover rates between resting and expanding cells^58,59^. While we cannot distinguish between these two effects, our data indicate that distribution of mCherry protein to daughter cells is likely to be a factor, because the total number of mCherry-expressing activated cells increases from 1 to 4–6 days post transfection (Fig. 6D) by a factor of about 2–3 before it starts to decrease. In the activated cells, we observed that the fraction of mCherry-expressing cells reduced by a factor of 2 after about 6 days post transfection. Using the ThermoFisher Neon bulk electroporation system to transfect primary human T cells with GFP-encoding mRNA, Aksoy et al.^60^ observed that the number of GFP-expressing cells decreased by a factor of 2 in 3–4 days post transfection. This decay was more rapid than that observed in our data, but this is not surprising, as many factors that can influence protein expression (the transfected gene, electroporation protocol, and mRNA concentration) differed between the two studies.

### High-throughput electrotransfection

We ran most of our optimization experiments using a cell concentration of 2 million/ml, which corresponds to a throughput of 0.8 million cells processed per minute, or 48 million per hour. However, cellular therapies often require more than 1 billion cells to be processed^61^, and there are major logistical advantages if this can be accomplished within a standard, 8-h work shift. This corresponds to minimum throughput of 125 million cells per hour, or 2.08 million cells/min. Furthermore, as manufacturing needs continue to grow and there is increasing development toward off-the-shelf allogeneic therapies^7,21^, billions of cells or more will likely need to be processed in minutes. We designed our microfluidic transfection system to meet or exceed these clinical-scale manufacturing requirements. To demonstrate high-throughput manufacturing, we tested our system at several higher cell concentrations, including 10, 20, and 50 million per ml (Fig. 5). We observed that performance at these cell concentrations remained high and comparable to our data collected using a cell concentration of 2 million per ml, with transfection efficiencies of approximately 70–75% for an mRNA concentration of 20 μg/ml, and efficiencies of 75–95% for an mRNA concentration of 50 μg/ml. At a concentration of 50 million cells per ml, the processing throughput was 20 million cells/min, or 1.2 billion cells in 1 h. This throughput can likely be increased by a factor of 2–4 by increasing the cell concentration further, another factor of 2–4 by running the system at a higher total flow rate, and by a factor of 10 or more by creating a system with 10 or more parallel microfluidic channels. In total, a factor 40–160 increase in throughput is possible. In addition to showing high throughput, we demonstrated that our device maintains high transfection efficiency (70–80%) over processing of large, clinically-relevant samples of activated T cells (200 million–500 million cells). Taking into account transfection efficiency, viability, and recovery, this corresponds to approximately 57% of the viable cells introduced into the system (averaged across donors) that were successfully recovered and observed to express the delivered gene.

At higher cell densities, fewer cargo molecules are available per cell, so we expected that using a higher cell concentration in our device would result in a decrease in transfection efficiency. We aimed to compensate for this decrease by additionally testing these higher cell concentrations with a higher concentration of mRNA, as informed by our mRNA dose-response data (Fig. 3A). Indeed, we observed higher transfection efficiency when the mRNA concentration was 50 μg/ml when compared to 20 μg/ml at all of the cell concentrations tested. However, as the cell concentration increased, the difference in transfection efficiency between the 20 and 50 μg/ml conditions decreased. This suggests that transfection efficiency may not be exclusively governed by the availability of cargo molecules, but additionally by the kinetics governing the probability of interaction between the cells and the cargo, which increases with both increased cell concentration and cargo concentration.

### Comparison to other electrotransfection systems

Most commercial electroporation systems designed for transfection of primary mammalian cells (e.g., Lonza Nucleofector 4D, ThermoFisher Neon) use cuvettes for bulk, batch processing. These systems can provide excellent performance, but are challenging to scale for clinical production and are difficult to integrate into an automated workflow. Flow-based electroporation has several advantages, including increased throughput^62^, greater potential for automation and integration, and improved thermal dissipation. The MaxCyte GT is one example of a commercial system that uses flow-based electroporation to increase throughput by orders of magnitude.

Though many microfluidic flow-based electrotransfection systems have been developed^12,23,24^, few have been optimized for primary mammalian cells, and most have not demonstrated transfection of clinically-relevant sample sizes. Microfluidic systems have advantages of increased precision, favorable thermal profiles related to high surface area to volume ratio^63^, and lower voltage requirements for electrical stimulation^64^. However, these advantages often come at the cost of throughput, making the transition to clinical-scale processing more challenging. Here we demonstrated truly continuous-flow processing of clinically-relevant sample sizes (500 million cells) in a microfluidic device with no major loss in transfection performance. The sample sizes tested in this study were limited only by the number of cells obtainable from a single buffy coat donor. We did not observe any signs of device degradation or failure, so we anticipate that significantly larger numbers of cells can be transfected in a single run. Furthermore, these samples were processed in 20 min or less, but even faster processing is possible by increasing flow rate and/or cell density.

### Conclusions, future work, and impact

Here we demonstrated clinical-scale gene delivery to primary human T cells using a microfluidic device in a truly continuous process. We transfected samples containing 200–500 million cells with mRNA at high efficiency and with minimal impact on viability, and showed that transfection did not limit expansion potential. As electroporation emerges as a prominent gene-delivery method in modern and novel cellular therapy manufacturing workflows^13,20,65,66^, high-throughput, large-scale, automated electrotransfection platforms will be critical for meeting production demand. In particular, moving from autologous treatments that require doses of hundreds of millions to billions of cells, toward allogeneic off-the-shelf therapies that aim to scale to rapid generation of trillions of therapeutic cells^28^, will likely require multiplexed delivery of a combination of payloads and payload types. Our device is well-positioned to meet these throughput and scaling needs, and to maximize impact for cellular therapy manufacturing, future work will focus on optimizing delivery of additional payloads, including RNPs and pDNA.

## Materials and methods

### Materials and reagents

Sheets of polyetherimide (PEI) (thicknesses including 127 μm, 254 μm, and 2.38 mm) and 25-Ga stainless steel tubing (508 μm OD; 254 μm ID) were obtained from McMaster Carr (Robbinsville, NJ). Sheets of PEI 25.4 μm in thickness were purchased from Sigma Aldrich (St. Louis, MO). R/flex 1000 films (12.7 μm thick) used for bonding polymer layers together were obtained from Rogers Corporation (Chandler, AZ). EPO-TEK 353ND epoxy (Epoxy Technology, Billerica, MA) was used to seal stainless steel tubing at the device inlets and outlets. Medical grade micro vinyl tubing with 0.38 mm inner diameter and 1.09 mm outer diameter was purchased from Scientific Commodities (Lake Havasu City, AZ).

T cells were isolated by Ficoll centrifugation followed by negative magnetic selection from buffy coat samples purchased de-identified from healthy human donors (see Isolation and culture of primary human T cells section below for additional details). For all device experiments, cells were suspended in BTXpress electroporation media (BTXpress Cytoporation Low Conductivity Medium T, BTX, Holliston, MA, USA) and RPMI 1640 culture medium with no glutamine and no phenol red (ThermoFisher) was used as the sheath fluid.

### Experimental procedure

In preparation for each device experiment, T cells were washed with PBS, resuspended in BTXpress low-conductivity electroporation media at a concentration of 2 million cells per ml, and mixed with CleanCap mCherry mRNA (TriLink Biotechnologies) at the indicated concentrations for each experiment. These cell-mRNA mixtures were loaded into syringes. The microfluidic device was sterilized by autoclave and transferred to a biosafety cabinet containing a sample stage, camera, two syringe pumps, and microcentrifuge tubes for sample collection. Cell suspensions were flowed continuously through the central inlet of the electroporation device at a flow rate of 400 μl/min using a syringe pump (PHD Ultra, Harvard Apparatus, Holliston, MA). High-conductivity cell culture buffer (RPMI) was flowed into the two side inlets of the device, at a rate of 550 μl/min per side, using a second syringe pump. The combined fluid flow rate through the device was 1500 μl/min.

At the beginning of each experiment the relative outlet flow fraction (amount of fluid leaving through the side outlets versus the central outlet) was set to 2 (1000 μl/min total out the sides versus 500 μl/min out the center) by adjusting the hydrodynamic resistances at the device outlets (trimming the outlet tubing). The side and center stream outputs were collected in microcentrifuge tubes and their masses were determined using a precision balance. These measurements were repeated periodically throughout the duration of experiments to ensure consistency of the outlet flow fraction.

Throughout each electroporation experiment a custom LabVIEW interface running on a PC was used in conjunction with a custom in-house built amplifier (see Supplementary Information for additional details) to deliver monophasic square-wave electroporation pulses across the microfluidic device electrodes. The software enabled user control of the amplitude of the applied pulse, $\text {V}_o$, the pulse duration, $\tau _{\text {pulse}}$, and the pulse frequency, *f* (Fig. 1C). Pulses were continuously delivered as cells flowed through the microfluidic device and the electric-field-containing fluid region. Following electroporation, cells were counted (for recovery measurements) and plated at a density of 1 million cells per ml in TexMACS Medium, a high-ionic-strength medium, containing 100 U/ml Human IL-2. Transfection efficiency and cell viability were measured the following day (unless otherwise indicated) by flow cytometry.

### Isolation and culture of primary human T cells

T-cell isolation was performed as described in our previous work^32^. De-identified, unpurified buffy coats from healthy human donors were obtained from Research Blood Components (Watertown, MA). Peripheral blood mononuclear cells (PBMCs) were isolated by Ficoll centrifugation in Ficoll-Paque PLUS (1.077 g/ml) density gradient media (GE Healthcare) according to the manufacturer’s instructions. T cells were isolated from the PBMCs by negative selection using a Pan T Cell Isolation Kit (Miltenyi Biotec) per the manufacturer’s instructions. Purified T cells were frozen using Recovery Cell Culture Freezing Medium (ThermoFisher) and stored in liquid nitrogen until needed. For experiments that required unstimulated resting cells, frozen T cells were thawed 24 h prior to an experiment using a 37 °C water bath, and cultured at an approximate density of 1 million per ml in TexMACS Medium (Miltenyi Biotec) containing 100 U/ml Human IL-2 (Miltenyi Biotec). For experiments that required activated cells, frozen T cells were thawed and activated 48 h prior to an experiment using a 37 °C water bath, and cultured at an approximate density of 1 million per ml in TexMACS Medium containing 100 U/ml Human IL-2 and a 1:100 dilution of T Cell TransAct (Miltenyi Biotec) activation beads.

### Cell counting and recovery measurements

Cells were mixed 1:1 with Trypan Blue and counted in Countess Cell Counting Chamber Slides using a Countess II FL Automated Cell Counter (ThermoFisher). Two cell counts were obtained and averaged for each sample. Absolute cell counts were obtained by multiplying the measured cell concentration by the sample volume, and Recovery was calculated by dividing the absolute cell count of the output sample by the absolute cell count of the input sample.

### Flow cytometry

Flow cytometry was performed as described in our previous work^32^. T cell samples were pelleted, washed, and resuspended in autoMACS Rinsing Solution (Miltenyi Biotec) containing 1:20 dilution of MACS BSA Stock Solution (Miltenyi Biotec) and 1:1000 Sytox Live/Dead stain (ThermoFisher) as per manufacturer’s instructions. Flow cytometry was performed on an Attune NxT Acoustic Focusing Cytometer (ThermoFisher). Data were analyzed in FlowJo V10. To measure transfection efficiency of mCherry mRNA: T cells were gated in FSC-A/SSC-A plots to exclude dead cells and cell debris, followed by gating on single cells in FSC-A/FSC-H plots, then gating on live cells that were Sytox Blue negative, and finally gating the mCherry positive and negative populations (Figure S3A). To measure viability: cell debris was excluded in FSC-A/SSC-A plots, followed by gating on single cells in FSC-A/FSC-H plots, and finally gating on the Sytox Blue negative and positive populations, representing the live and dead cells, respectively (Figure S3B).

### Computational modeling

Electric fields generated in our device were estimated using a finite element analysis model implemented in COMSOL Multiphysics 5.4 software that sequentially solved for steady-state fluid flow, ionic species transport, and electric currents in three dimensions. The Reynolds number in the system at the flow rates tested was of order 10, so the Navier-Stokes equations for laminar, incompressible flow were used to solve for the fluid velocity field. All fluids in the device were assumed to have the density and viscosity of water at 25 °C. Laminar inflow boundary conditions were set for the sheath stream inlets, with flow rates of 550 μl/min for each. A similar condition was used for the center stream inlet, but with a flow rate of 400 μl/min. A zero gauge pressure boundary condition was applied to all three outlets. All other surfaces in the channel had a no-slip boundary condition. In the next solution step, the steady-state velocity solution was used in solving for ionic species transport. Because the bulk solution conductivity can be expressed as a linear combination of constituent ionic species, we treated the conductivity as the transported quantity and solved for its distribution directly. One of the medias used, BTXpress, is proprietary, so we assumed that all ionic species in solution were small molecules, and used a diffusion coefficient of 1 × 10$^{-9}$ m$^2$ s$^{-1}$. For the side outlets, we applied inflow boundary conditions where the conductivity was set to 14 mS cm$^{-1}$, the approximate conductivity of RPMI. For the center inlet, we set the conductivity for the inflow condition to 0.5 mS cm$^{-1}$, the approximate conductivity of cells and mRNA suspended in BTXpress media. Outflow boundary conditions for conductivity transport were applied at the outlets. Finally, the steady-state conductivity distribution was used to solve for the electric field magnitude distribution. An electrical potential of $V_o/2$ was set as the boundary condition on one electrode, and a potential of $-V_o/2$ was set on the counter electrode, where $V_o$ is the applied potential, which varied in the range 0–70 V in this study. All other surfaces were treated as electrically insulating. In this simulation, we assumed that the charge-transfer resistance at the electrodes was negligible, the applied voltages were large enough to pass Faradaic current via electrochemical reactions at the electrode-electrolyte interface, and that the delivered pulses were long in duration compared to the electrical double layer charging time. Thermal effects were not considered, as Joule heating was not expected to generate a significant temperature rise at the average power levels tested. This is supported by our impedance measurements (Fig. 4C) in which the measured peak currents as a function of applied voltage agree well with those predicted by our computational model.

### T-cell expansion and growth rate measurement

T cells were transfected on Day 0 and cell count, mCherry expression, and viability were measured on Days 1, 4, 6, 8, 11, and 13. On measurement days, T cells were counted and total sample volume was determined in order to calculate the absolute cell count. A 50-μl sample volume was used on each measurement day for flow cytometry analysis. To maintain a cell concentration of 1 million/ml, T cell sample volume was increased with the addition of fresh TexMACS Medium containing 100 U/ml Human IL-2 (Miltenyi Biotec). As volume increased, samples were transferred to larger culture vessels as necessary.

### Ethics approval and informed consent

All experimental protocols and the use of human-derived cells were reviewed and approved by Draper’s Institutional Biosafety Committee (institutional and/or licensing committee), approval number 13121218OR. All methods were carried out in accordance with the guidelines and regulations approved by Draper’s Institutional Biosafety Committee. All human blood products were purchased from Research Blood Components. Research Blood Components obtains IRB-approved informed consent from all donors giving permission to collect their blood or blood product and use or sell it for research purposes.

## Supplementary information

Supplementary Information.
