# Supplementary material for: High-throughput continuous-flow microfluidic electroporation of mRNA into primary human T cells for applications in cellular therapy manufacturing
Source: Sci Rep. 2020 Oct 22;10:18045. doi: 10.1038/s41598-020-73755-0 (PMC7582186; doi:10.1038/s41598-020-73755-0)
Supplement: Supplementary file 1 — Supplementary Information. [file 41598_2020_73755_MOESM1_ESM.pdf]

High-Throughput Continuous-Flow Microfluidic Electroporation of mRNA into Primary Human T Cells for Applications in Cellular Therapy Manufacturing – Supplementary Information

Charles A. Lissandrello[1], Jose A. Santos[1], Peter Hsi[1], Michaela Welch[1], Vienna L. Mott[1], Ernest S. Kim[1], Jordan Chesin[1], Nerses J. Haroutunian[1], Aaron G. Stoddard[1], Andrew Czarnecki[1], Jonathan R. Coppeta[1], Daniel K. Freeman[1], Deborah A. Flusberg[1], Jenna L. Balestrini[1], and Vishal Tandon[1,*]

[1]The Charles Stark Draper Laboratory, Inc., Cambridge, MA 02139, USA
[*]Correspondence and requests for materials should be addressed to V.T. (email: vtandon@draper.com)

Electronic Supplementary Information

Supplemental Methods

Device Fabrication

The electroporation device comprises a multi-material stack of several patterned sheets that are laminated together to form a three-dimensional microfluidic network (Figure S1), as has been described in detail in previous work[1–3]. Once a channel design was optimized computationally, features were defined using computer-aided design (CAD) software (Dassault Systemes Solidworks). Next, these features were machined into sheets of polyetherimide (PEI) of varying thickness (25.4 μm, 127 μm, 254 μm, and 2.38 mm). Each layer was first baked at 175°C for 18 hours to relieve stress in the material. The 2.38 mm sheets required a 25-pound weight to be placed on top during the baking process to prevent any deformation of the material. Next, an adhesive (R/flex 1000, Rogers, Chandler, AZ) was tacked to some of the layers by applying $\sim$ 250 kPa of pressure at 100°C for 10 minutes in a heated press (Carver 3895 Automatic Hydraulic Laboratory Press, Carver, Wabash, IN). Finally, the PEI layers were precision-cut using an ultraviolet laser source (LPKF ProtoLaser U4, LPKF Laser & Electronics, Garbsen, Germany) or by conventional CNC milling (2.38 mm layer only).

The PEI sheet that formed the electrode layer was patterned with metal by using a sputter deposition system (KDF 954ix). First, the PEI sheet was cleaned in an ultrasonic bath in a 1:1 solution of deionized water and isopropanol. Next, the PEI and a patterned shadow mask, which defined the geometry of the device electrodes, were aligned and loaded into the sputtering tool. The surface of the PEI (matte side) was cleaned and roughened in-situ prior to deposition, by exposing it to argon plasma for 10 minutes (200 W, 3 mTorr). The tool was then used to deposit a 100-nm thick layer of titanium followed by a 200-nm thick layer of platinum through the shadow mask onto the PEI surface. The patterned metal formed the electrodes, which made contact with the fluid inside the microfluidic channel.

All device layers were cleaned by treating with oxygen plasma (PX-250 Plasma Chamber, March Instruments, Concord, MA) for 30 seconds per side (100 W). The layers were then assembled in a two-step lamination procedure. First, the inlet/outlet interface plate, the electroporation electrode layer, and the electrode mask layer (layers 1, 2, and 3 in Figure S1) were aligned using 1/16"-diameter stainless steel dowel rods inserted into alignment holes and laminated together in a custom-built chamber at high temperature and pressure (175°C and $\sim$ 2000 kPa for one hour). After allowing time for cooling, the microfluidic channel layer and the cover layer (layers 4 and 5 in Figure S1) were added and the entire stack was placed back in the laminator for the second lamination step (175°C and $\sim$ 2000 kPa for one hour). The assembled stack was removed from the laminator and a 1-cm length of 25-gauge stainless steel tubing was inserted into each port opening. A two-part epoxy (EPO-TEK 353ND, Epoxy Technology, Billerica, MA) was then prepared and dispensed around the base of each piece of tubing to secure the pieces into the microfluidic device. The epoxy was cured in an oven overnight at 60°C. Finally, before use in an experiment, each completed device was sterilized by autoclave (120°C for 30 minutes) and medical grade micro vinyl tubing (0.38 mm inner diameter and 1.09 mm outer diameter) was attached to each inlet and outlet.

Electronics and software control

A custom waveform generation system was designed and built to generate and monitor electroporation waveforms. The system comprised a multifunction data acquisition (DAQ) system (National Instruments USB-6361) with analog inputs and outputs, two DC power supplies (Sorensen XPH 75-2D), a custom linear amplifier (based on the Apex PA96 operational amplifier) with built-in current and voltage monitoring, and a PC running a custom interface built in LabVIEW for waveform control and monitoring. The interface allowed the user to define waveform parameters including the pulse voltage, duration, and repetition rate. The prescribed waveform was generated at an analog output of the DAQ and then amplified using the linear amplifier. The amplifier had maximum voltage and current output capabilities of 70 V and 680 mA, respectively, and was powered by two DC power supplies providing +75 VDC and -75 VDC. The amplifier included built-in current sensing circuitry that monitored the voltage drop across a sense resistor configured in series with the electrical load. The amplifier also included a voltage divider circuit that provided a voltage output which was linearly proportional to the instantaneous voltage delivered to the electrical load. These two signals were measured using analog inputs on the DAQ and were recorded by the computer.

Statistical Analysis

A 4-factor analysis of variance (ANOVA) was performed using MATLAB r2018b software. The effects of applied voltage, pulse duration, number of pulses, and donor on transfection efficiency, viability ratio, and recovery were analyzed for all of the data in Figure 3, where each data point was an average of 3 donor replicates (Table S1). Here the "donor" factor includes variation due to the cell donor, device-to-device variability, and the day on which the experiment was performed, because these factors cannot be decoupled. All other statistical analyses were performed in GraphPad Prism 8 software, including multiple t tests to analyze the data in Figure 5.

References

1. Mescher, M. J. *et al.* Fabrication methods and performance of low-permeability microfluidic components for a miniaturized wearable drug delivery system. *J. Microelectromechanical Syst.* **18**, 501–510, DOI: 10.1109/JMEMS.2009.2015484 (2009).
2. Tandon, V. *et al.* Microfabricated infuse-withdraw micropump component for an integrated inner-ear drug-delivery platform. *Biomed. Microdevices* **17**, 3–21, DOI: 10.1007/s10544-014-9923-8 (2015).
3. Tandon, V. *et al.* Microfabricated reciprocating micropump for intracochlear drug delivery with integrated drug/fluid storage and electronically controlled dosing. *Lab Chip* **16**, 829–846, DOI: 10.1039/C5LC01396H (2016).

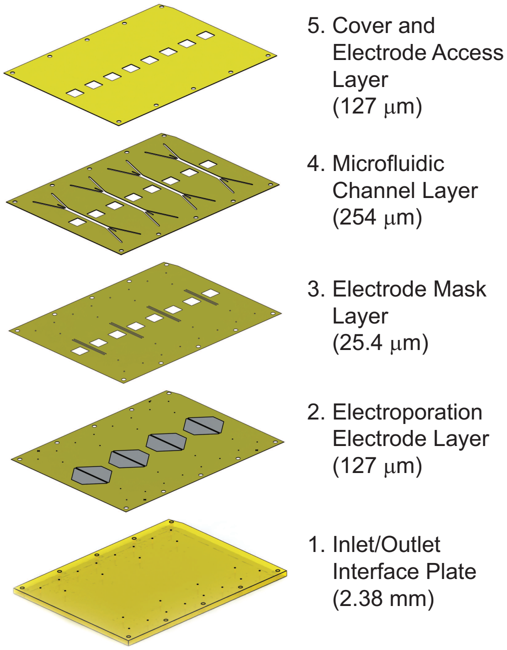

Figure S1. Scheme of the polymer layers that comprise our microfluidic device. Layer 1 is machined using a CNC milling machine at Draper's machine shop. The other layers are cut using a UV laser. Platinum electrodes are sputtered onto layer 2 using a shadow mask process. A thin-film adhesive interspersed between the layers is used to bond them together during lamination.

Table S1. Multifactor ANOVA analysis of the effects of electrical parameters and T-cell sample donor on cell viability ratio (the data shown in Figure 3), transfection efficiency, and recovery for resting primary human T cells transfected with mRNA in our device. This analysis was carried out in MATLAB r2018b software. Here, the term, "Donor," includes the effects of the T cell sample donor, the particular microfluidic device used, and the day on which the experiment was done. There were three replicates (i.e., three independent, healthy T-cell donors) for each condition.

**Factor**	**Degrees of Freedom**	**Viability Ratio p Value**	**Transfection Efficiency p Value**	**Recovery p Value**
Applied Voltage	4	0	0	0.8374
Pulse Duration	2	0.0001	0.0008	0.0453
Number of Pulses	2	0.0237	0	0.00452
Donor	2	0.0361	0.1219	0

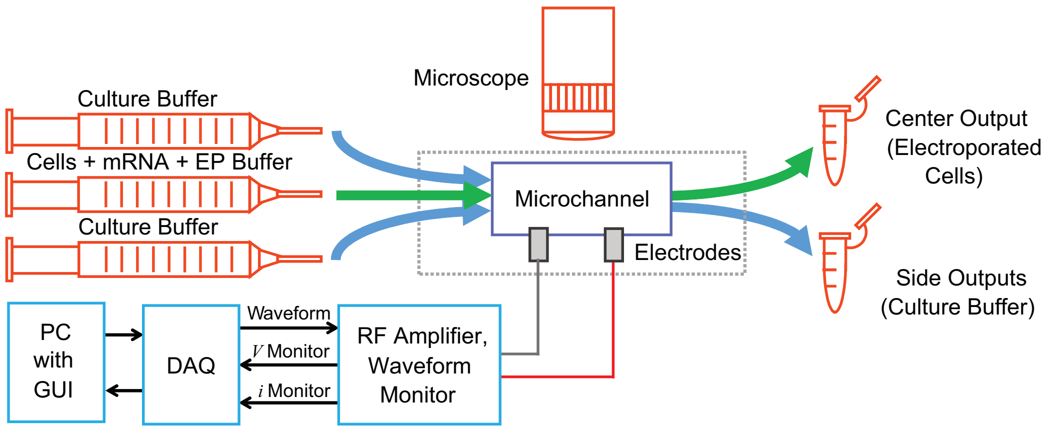

Figure S2. Block diagram of the system and control electronics. The custom microchannel with integrated electrodes sits on a microscope stage. A computer running control software communicates with a DAQ, which generates the electroporation waveform and reads out the instantaneous voltage and current delivered across the electrodes. A custom electronics box amplifies the waveform and conditions the voltage and current signals for readout by the DAQ. High-conductivity culture buffer and low-conductivity electroporation buffer (containing cells and mRNA) are delivered at a controlled rate to the sheath and cell inlets, respectively, using multiple syringe pumps. Relative sheath vs. center flow fractions are controlled by adjusting the hydrodynamic resistances at the outlets using appropriate lengths of outlet tubing. The side and center stream outputs are collected in microcentrifuge tubes for further analysis.

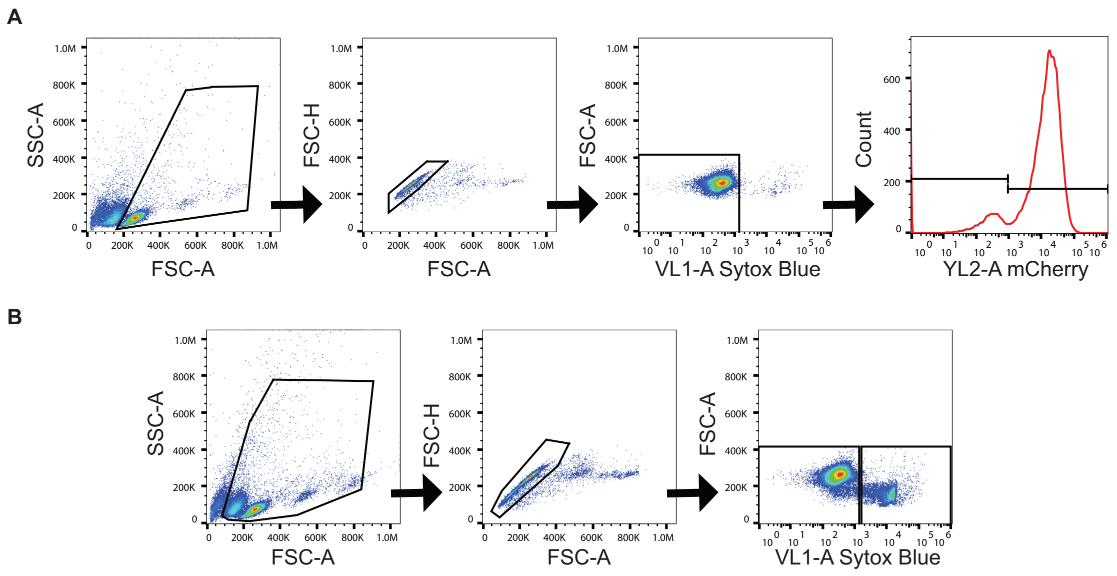

Figure S3. Representative flow cytometry plots showing the gating strategy used to determine (A) mRNA transfection efficiency, and (B) viability at 24 hours.
